# Functional outcome and complications after treatment of comminuted tibial fractures or deformities using Ilizarov bone transport: a single-center study at 15- to 30-year follow-up

**DOI:** 10.1007/s00402-020-03562-9

**Published:** 2020-07-30

**Authors:** Carlo Biz, Alberto Crimì, Ilaria Fantoni, Marco Vigo, Claudio Iacobellis, Pietro Ruggieri

**Affiliations:** Department of Surgery, Oncology and Gastroenterology DiSCOG, Orthopaedic, Traumatological and Oncological Clinic, University of Padua, via Giustiniani 2, 35128 Padua, Italy

**Keywords:** Bone transport Ilizarov, Distraction osteogenesis, External fixation, Tibial deformity, Tibial fracture

## Abstract

**Introduction:**

The aim of this retrospective study was to evaluate long-term outcomes and complications of a single-center and single-surgeon patient series of isolated and comminuted tibial fractures with bone defects or tibial deformities treated by Ilizarov bone transport.

**Materials and methods:**

Data from a consecutive series of patients with isolated comminuted tibial fractures (*Fracture Group: FG*) or deformities (*Deformity Group: DG*) treated between 1987 and 2002 were included. For clinical assessment, the Lower Extremities Functional Scale was used; complications were recorded according to the Dindo classification and statistical analysis was performed.

**Results:**

Overall, 72 patients were enrolled with a mean follow-up of 21.6 years (range 15–30) a mean LEFS of 36.4 (range 0–100). In the *FG*, the mean LEFS was 21.3 (range 0–98.75), and the external fixation time (EFT) lasted 7.6 months (range 3–18 months) months. In the *DG*, the mean LEFS was 76.7 (range 55–100), and the EFT was 10.6 months (range 3–20 months). Between the two groups, the clinical evaluation was significantly different, while the EFT was not (*p* = 0.14). In the *FG*, the worst results were obtained in the cases of open fractures with a higher percentage of complications and the need for further surgical procedures. The cumulative rate of complications was 55.6% during the first 36 months and 66.7% at the minimum follow-up of 180 months.

**Conclusions:**

Ilizarov bone transport, even at a long follow-up period, proved to be an effective technique for both definitive treatment of comminuted tibial fractures with bone defects or tibial deformities. Although our functional outcomes were lower in patients with exposed fractures, they were in line with the literature, but not influenced by the EFT when properly managed. Most complications occurred during the first 3 years; however, they could also arise much later, even until almost 30 years.

## Introduction

The treatment of tibial bone and soft-tissue defects, especially greater than 5 cm, secondary to high-energy trauma or debridement for non-union, are great challenges for the orthopaedic surgeons in terms of limb reconstruction and soft tissue coverage. The management of these injuries has been revolutionised so much by the Ilizarov method that some authors have declared this technique of distraction osteogenesis the gold standard for bone tibial defects [1, 2]. Ilizarov bone transport is useful in a wide number of other clinical conditions, both in paediatric and adult age [3, 4], even if the most common use remains the treatment of limb length inequality or complex deformities.

Although tibial bone defects treated by Ilizarov methods have reached satisfactory outcomes in most studies, there have still been some relatively unsatisfactory results in several reports [3, 5]. As gradual lengthening of the tibial bone usually requires long-term external fixation time (EFT), minor and major complications are very common and some of them may persist years later. The most frequent ones are pin-track infection, malunion, refracture, joint contracture and stiffness, nerve palsy, decreased motion of the joints and paraesthesia [6]. To prevent them, other methods have been devised for tibial bone transport, such as mono-lateral fixators or intramedullary nail systems [7].

Although Ilizarov fixators have been in use for many years, very few reports have focused on their long-term outcomes in tibial bone defects or deformities [8]. Hence, the aim of this retrospective cohort study was to evaluate long-term outcomes and complications of a single-center and single-surgeon patient series with isolated and comminuted tibial fractures with bone defects or tibial deformities treated by Ilizarov bone transport.

## Materials and methods

### Patients

This study included clinical and radiographic data from a consecutive series of Caucasian patients with a diagnosis of isolated comminuted tibial fractures with bone loss *(Fractures Group: FG)* or tibial deformities *(Deformities Group: DG)* treated by the Ilizarov External Fixator between 1987 and 2002. For several years, our level-I healthcare trauma center, a multi-specialty regional university teaching hospital, has been authorised to use Ilizarov frames routinely. All subjects participating in this study received a thorough explanation of the analysis and gave their oral and written informed consent to publish the data. The study was performed in accordance with the ethical standards of the 1964 Declaration of Helsinki as revised in the last and those of Good Clinical Practice and it was approved by the Local Ethical Committee (Prot. Num. 7764, Tit. II Cl.10 fasc.4).

### Inclusion and exclusion criteria

The inclusion criteria were adult patients at the time of surgery treated for isolated comminuted tibial fractures or tibial deformities by an Ilizarov external fixator during a minimum 3-month period, able to adequately answer a questionnaire for clinical evaluation after a minimum 15-year follow-up. In contrast, those subjects previously treated using either intramedullary nails or plates transferred to our institution from elsewhere, patients with a diagnosis of pathological fractures, primary arthrodesis or primary amputation, previous history of complete peroneal and tibial nerve palsy were excluded.

### Surgical technique and post-operative care

These procedures were performed according to the traditional Ilizarov bone transport method [9, 10] by the senior author with the help of two different residents of our institution. All patients underwent spinal anaesthesia, as this surgery does not require more than 2 h of operation for experienced surgeons when the fixator is assembled previously. They were then placed on a radiolucent operating table in the supine position. Antibiotic intravenous prophylaxis was administered with Cefazolin (1 g 4 times/day) and continued 24 h after surgery in cases of tibial closed fractures or deformities, while Ampicillin and Sulbactam (3 g 4 times/day) were administered for a week in cases of bone exposure. In cases of exposed or infected fractures, once the results of intraoperative cultural exams were available, the antibiotic therapy was adapted according to sensitivities and lasted for 6 weeks. Partial weight bearing using crutches was allowed during the first week, while full weight bearing as tolerated was permitted from the second week and continued during the transport bone period until consolidation. Postoperative antithrombotic therapy (Natrium Enoxaparin) was given only until full weight bearing. Systems and procedures were carried out according to fracture or deformity types.

In the *FG,* all cases of open fractures were subjected to thorough and accurate debridement to remove all of the devitalized tissues before applying the circular external fixator. The standard technique consisted of 6 rings: 2 proximal rings, 2 distal rings and 2 more rings, used for bone transport. Two 1.8 mm Kirschner wires were inserted and put in tension for each ring; 2 screws coated with hydroxyapatite were applied to the ring used for transport on the medial subcutaneous surface of the tibia to avoid crossing the muscle tissue of the tibia lateral side, reducing skin problems from the other crossed wires. For proximal or distal articular fractures, only one ring was used in the articular surface. K-wires with olive-stops were used to maintain the fracture reduction, which is essential, although in comminuted fractures, this was not always possible. In the *DG*, only one proximal, distal and another ring for bone transport were used in cases of hypometric tibias. In both groups, the osteotomy was performed with a Gigli saw after the fixator was applied. Bone transport started 5 days after osteotomy at an average rate of 1/4 mm every 6 h. Subsequently, since the end of 80 s, we started evaluating it by ultrasound exams, which usually shows non-mineralized new bone formation 2 months before radiographic confirmation. These exams were taken on the 10th and 20th days after the beginning of distraction, and in cases of cysts in the regenerated bone, 1 or 2 more ultrasounds were repeated every 15–20 days [11]. Daily care of the pins was recommended with the use of local disinfection, and oral antibiotics were given in case of pin-track infection or skin abscess. Bone grafting at the docking site was routinely performed to remove the exuberant scar tissue interposed and to promote the contact between the two irregular bone surfaces. Finally, the Ilizarov frame was kept until 2/3 of the circular cortical bone was visible on antero-posterior and lateral X-ray views, determining the time for frame removal.

### Patient assessment

Data collection was retrospectively performed by two external and independent investigators, young residents of our University Orthopaedic Clinic, not involved in the patients’ treatment. Consulting the computerized archives of our hospital, the following information was recorded for each group (if appropriate): patients’ age; gender; American Society of Anaesthesiologists (ASA) class of globally estimated surgical risk; neurovascular deficit after trauma; fracture mechanism, pattern and side; length of the gap as measured between the proximal and distal main bone where intact bone in all of the circumference was present (only for *FG*)*;* type of deformity (only for *DG*); time until bone union starting from the time of application of the Ilizarov fixator (EFT); type of distraction (single or double transport); and complications. The types of fractures were classified according to the AO/OTA system and the open ones according to the Gustilo-Anderson criteria.

At the time of this study, phone contact was attempted for all patients who met inclusion criteria. Patients were asked to fill out the Lower Extremities Functional Scale (LEFS) in their mother language. LEFS is reported to be a reliable self-evaluation; it is a well-known and validated patient-rated outcome measure (PROM) used to assess lower extremity function [12], including 20 activities for which the patients give a score between 0 (extreme difficulty or inability) to 4 (no difficulty) to perform each specific activity. Moreover, for each patient, complications and complaints were recorded and classified according to the Dindo classification [13]. Amputation was considered to be a zero LEFS score.

### Statistical analysis

Categorical variables were reported as a number of patients and percentage. Quantitative variables are summarised with mean and range. Normality of quantitative variables was evaluated by a box-plot and the Shapiro–Wilk test. Comparison of not normal quantitative variables between the two groups was performed using the *U*-Mann Whitney test. Comparison of categorical variables between the two groups was done using *χ*2 or Fisher’s exact test. Possible correlations between EFT and LEFS, complications or age at surgery were evaluated with Spearman’s Correlation Coefficient. Statistical analysis of data was performed by an independent statistician using SPSS statistical software (version 25.0; IBM). Statistical significance was considered for *p* < 0.05.

## Results

### Patients’ data

**Fig. 1:**
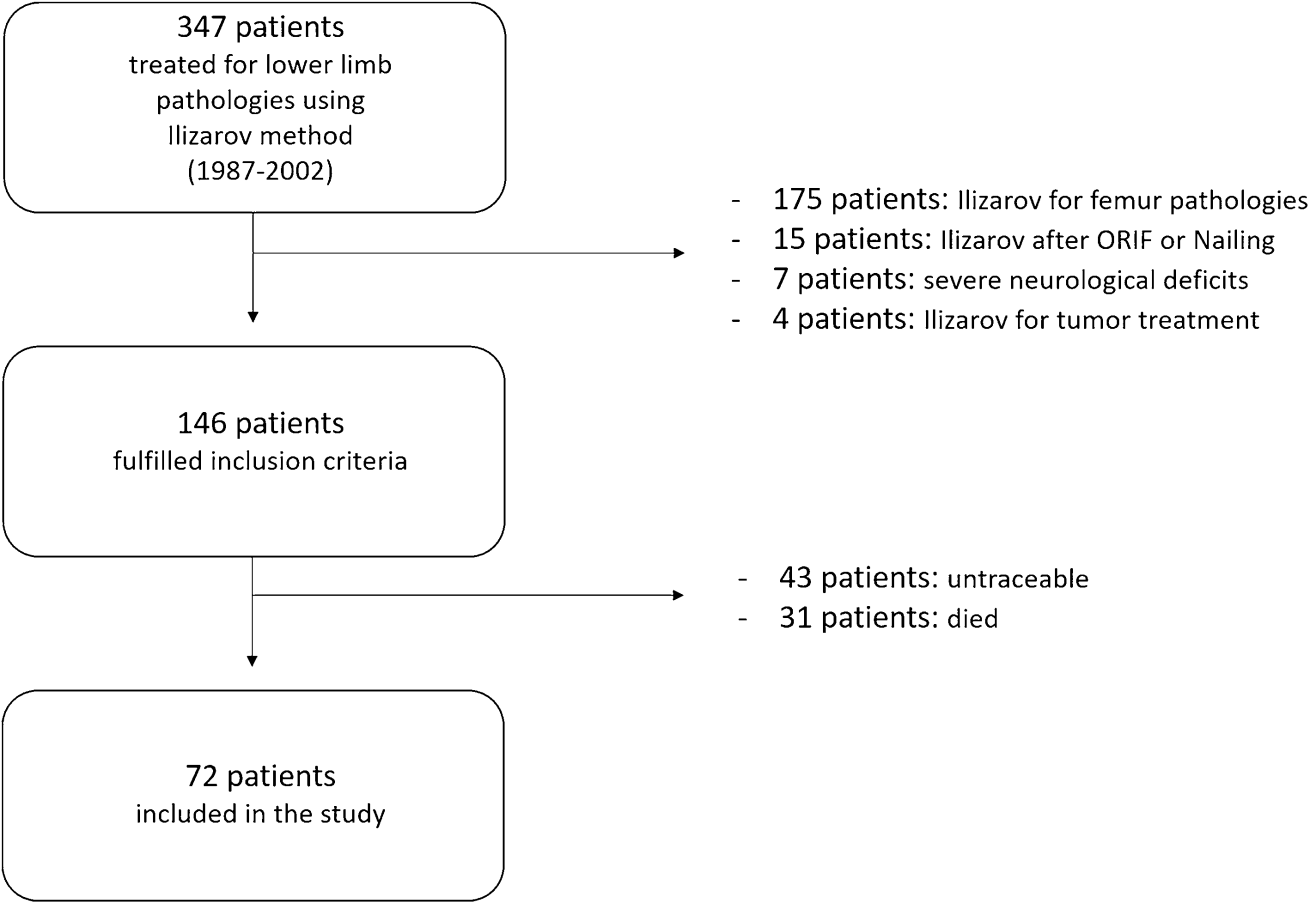
The flow-chart of patient selection according to inclusion and exclusion criteria of the study

Over a period of 15 years, 146 patients treated by Ilizarov technique at our institution and fulfilling the inclusion criteria were identified (Fig. 1). Among these, during the follow-up period, we could not evaluate 74 patients (50.7%) because of two main reasons: they could not be contacted by telephone (having moved), or they had died. Therefore, 72 among 146 (49.3%) were retrospectively enrolled in the present study; no one was lost up to the average final clinical follow-up of 21.6 years (range 15–30). The average patient’s age at the time of surgery was 50.2 ± 11.3 years (range 28–78) in the *FG* and 28.6 ± 10.5 years (range 16–50) in the *DG*. There were 46 males and 36 females. Sixty patients presented ASA 1 (83.3%), 11 ASA 2 (15.3%), 1 patient ASA 3 (1.4%).

**Fig. 2:**
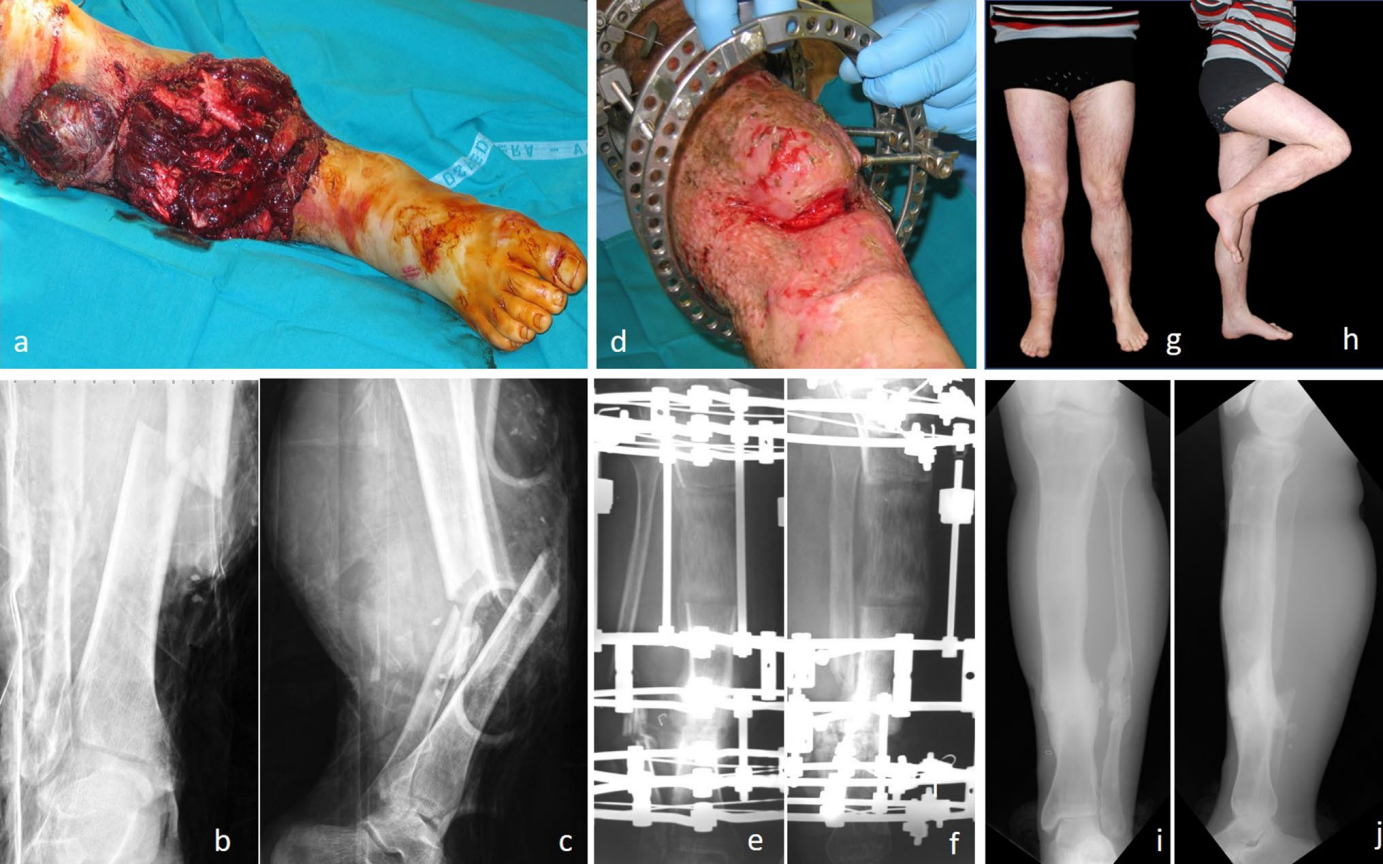
A 28-year-old male patient with an open fracture of the right tibia, Gustilo Anderson grade III-C with important bone and soft tissue defect: **a** right leg clinical presentation in operating room at arrival and preoperative X-rays on AP (**b**) and LL (**c**) views. Ilizarov bone transport: clinical image (**d**) and X-rays on AP (**e**) and LL (**f**) during treatment. Bone transport completed with good regenerate consolidation and docking union: clinical (**g**, **h**) and radiographic (**i**, **j**) images at 21-year follow-up

**Fig. 3:**
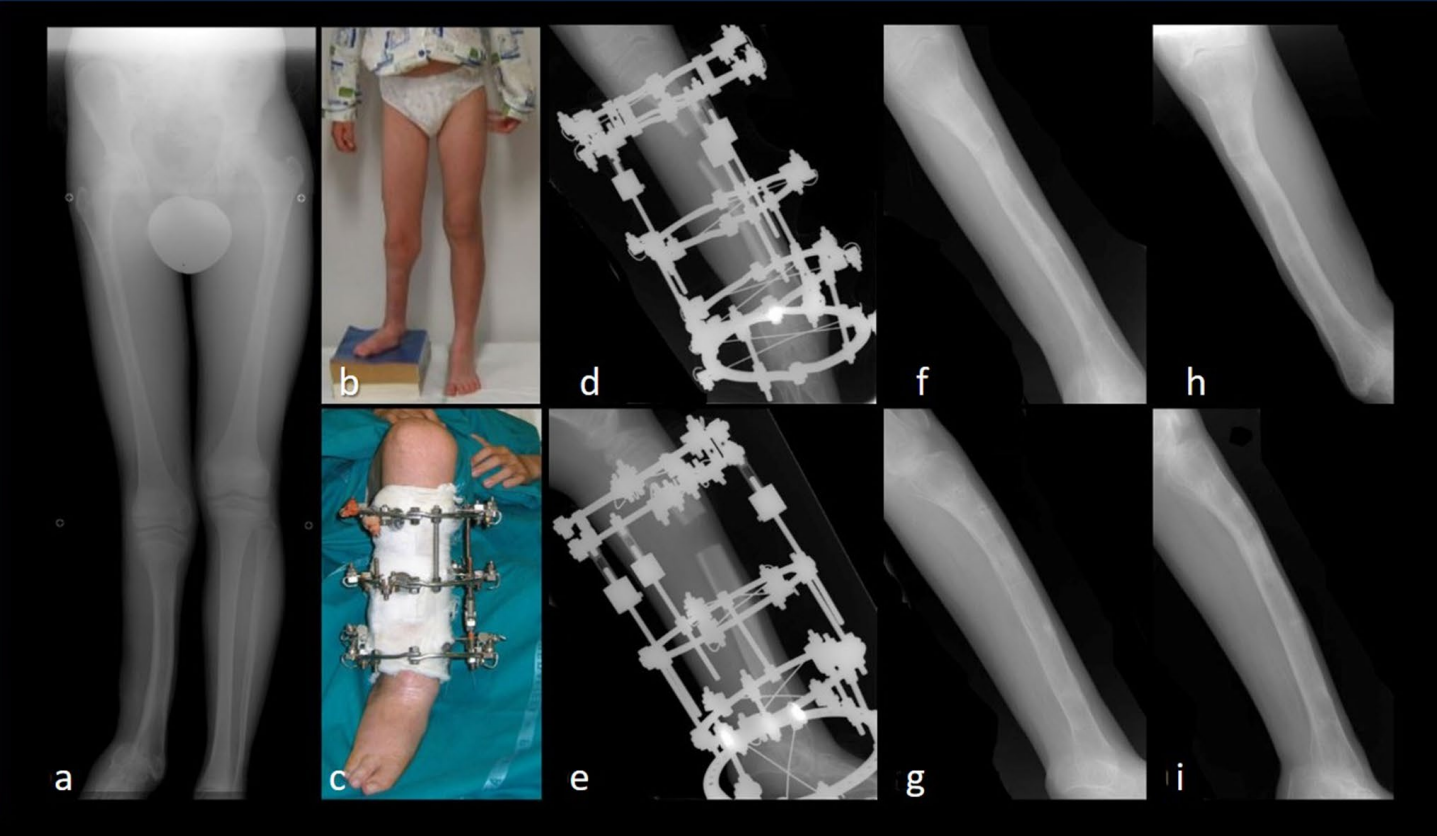
A 15-year-old patient affected by longitudinal lateral hemimelia of his right leg with complete absence of the fibula: preoperative AP X-ray (**a**) and clinical (**b**) images, showing 7-cm hypometria on the right lower limb. Ilizarov bone transport: clinical image (**c**) and X-rays on AP (**d**) and LL (**e**) during treatment. Radiographic results on AP and LL X-ray views after removal of the external fixator one year later (**f**, **g**) and at final follow-up of 18 months (**h**, **i**)

In the *FG*, there were 56 tibial comminuted fractures (Fig. 2), including 22 fractures type 41.A3, 41.B3 and 41.C3, 18 type 42.C3, 16 fractures type 43.A3, 43.B3 and 43.C3. In 42 cases (75%), the initial injury was an open tibial fracture as follows: 3 cases (7.1%) were Type II, 7 cases (16.7%) Type III A, 10 cases (23.8%) Type III B and 22 cases (52.4%) Type III C with either a neurological or vascular deficit. The right side was involved in 34 cases and the left side in 38. Regarding trauma, motor vehicle accident (MVA) was the most represented with 46 cases (82%), bicycle accidents in 6 cases (11%) and 4 cases (7%) of falls from height. In the *DG*, there were 16 patients treated, 6 cases for post-traumatic tibial deformities, 4 cases for valgus knees, 4 cases for hypometric tibias (Fig. 3) (1 case in an achondroplastic dwarf, for whom only one tibia was included in the follow-up) and 2 for tibial deformity after osteomyelitis. The EFT was 8.25 months (range 3–20). The mean bone union rate was 100%, as all patients, including those of *FG*, reached union by routinely receiving bone grafting. Finally, the mean lengthening achieved was 7.05 cm (range 4–15 cm). 65 patients (90%) received single transport; 7 (10%) received double site transport.

### Clinical outcomes

The mean LEFS was 36.4 (range 0–100). In the *FG*, the mean LEFS was 21.3 (range 0–98.75), and the Ilizarov procedure lasted for 7.6 months (range 3–18 months). In the *DG*, the mean LEFS was 76.7 (range 55–100), and the Ilizarov technique was applied for a mean period of 10.6 months (range 3–20 months). The EFT in the two groups was not statistically different (*p* = 0.14). The mean number of complications per patient was 0.9 (range 0–2). The clinical evaluation was significantly different in the two groups (*p* < 0.0001). There was no correlation between the EFT and the LEFS (*p* > 0.05). In *FG*, there was no correlation between EFT and LEFS (*p* > 0.05). Considering the overall population, a direct correlation was found between neurological deficit and open fracture (*R* = 0.561, *p* < 0.001), while an inverse correlation was found between neurological deficit and LEFS (*R* = − 0.559, *p* < 0.001). In the *FG*, direct correlations were found between neurological deficit and open fracture (*R* = 0.464, *p* = 0.013) and between EFT and open fracture (*R* = 0.495, *p* = 0.007). Moreover, an inverse correlation was found between neurological deficit and LEFS (*R* = − 0.537, *p* = *0.003)*.

No significative correlations were found in the *DG*. In the *FG,* for the patients with open fractures, the mean LEFS score was 18.6 (range 0–98.75), and the EFT was 8.6 months (range 3–18 months); for those with closed fractures, the mean LEFS score was 67.7 (range 40–98.75), and the EFT was 7.8 months (range 3–18 months). In these two subgroups (open and closed fractures), the difference in clinical outcomes was statistically significant (*p* < 0.0001), while EFT was not statistically significant (*p* = 0.7).

### Complications

**Fig. 4:**
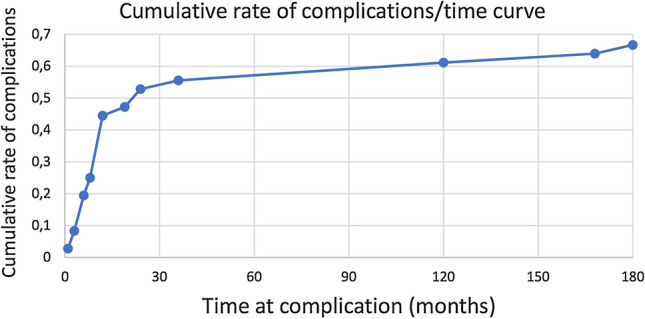
Cumulative rate of complications as a function of time at complication (in months); in the first 3 years, the majority of complications (55.6%) occurred with a following plateau reaching 66.7% of cumulative rate of complications at the minimum follow up of 180 months

Complications were reported in 56 (77.8%) of the 72 patients. Dindo classification type I complications not requiring surgical treatment were present in 42 patients (58.3%) (Table 1): knee stiffness (24 cases), leg paraesthesia or foot sensibility deficit (11 cases), peroneal nerve deficit (5 cases) and foot varus deformity (2 cases). These complications were reported over a long period of time (months from surgery to almost 30 years in the case of knee stiffness). Type II complications requiring pharmacological treatment were present in 4 patients (5.6%) with infections post-Ilizarov removal; these patients were treated with intravenous and oral antibiotics. No cases of type IIIa complications requiring surgical intervention without anaesthesia were reported. Type IIIb complications requiring further surgery under anaesthesia were present in 18 patients (25%): 5 cases of total knee arthroplasties (performed 1–16 years after Ilizarov surgery), 5 cases of ankle arthrodesis, Achilles tendon tenotomies in 4 cases, above the knee amputations in 4 cases (performed 1–10 years after Ilizarov surgery) due to the development of infections and to neurovascular impairment of the lower limbs. In *DG*, there were only Type I complications based on Dindo’s criteria. No patient suffered from compartment syndrome during treatment. The cumulative rate of complications in the first 36 months was 55.6%, followed by a plateau to reach a complication rate of 66.7% at the minimum follow up of 180 months (Fig. 4).

**Table 1:** Classification of surgical complications according to Dindo and sample population complications

Grade	Definition	Sample complications (Pts: patients)
Grade I	Any deviation from the normal postoperative course without the need for pharmacological treatment or surgical endoscopic, and radiological interventions. Allowed therapeutic regimens are: drugs as antiemetics, antipyretics, analgesics, diuretics, electrolytes and physiotherapy	24 Pts with knee stiffness; 11 Pts with leg paraesthesia or foot sensibility deficit; 5 Pts with peroneal nerve motor deficit; 2 Pts with foot varus deformity
Grade II	Requiring pharmacological treatment with drugs other than such allowed for grade I complications	4 Pts with infections post Ilizarov removal treated with endovenous and oral antibiotic
Grade III	Requiring surgical, endoscopic or radiological intervention	
Grade IIIa	Intervention not under general anaesthesia	
Grade IIIb	Intervention under general anaesthesia	5 Pts required total knee arthroplasty; 5 Pts required ankle arthrodesis; 4 Pts required Achilles tendon tenotomies; 4 Pts required above the knee amputation
Grade IV	Life-threatening complication requiring IC management	
Grade IVa	Single organ dysfunction	
Grade IVb	Multiorgan dysfunction	
Grade V	Death of a patient

## Discussion

The Ilizarov method for bone transport is a versatile technique, useful in different complex orthopaedic conditions. Several potential advantages are as follows: fracture reduction and fragment fixation can be achieved with almost no soft-tissue exposure or blood loss; the adjustment of the alignment with compression or distraction of the fracture fragments can be performed both during and after the primary surgical intervention; the fixator is stable enough to allow early weight bearing irrespective of the type of fracture; finally, no implants are left in situ when the fracture is healed [14]. Nevertheless, it is very labour-intensive for both patient and surgeon, requiring patient education, compliance, demanding learning curve, and specialised staff [15]. For these reasons, it should be managed only in specialised experienced centers, such as ours, with the ability to cope with the complications intrinsic with this procedure and its length of treatment [6]. Although several authors have shown the benefits of the Ilizarov system when dealing with soft tissue loss and comminuted fractures after high energy traumas, as well as skeletal deformities [4, 16], to the best of our knowledge, only 3 meta-analyses and systematic reviews have evaluated its effectiveness, complications and clinical results in the treatment of long bone tibial defects [5, 17, 18]. This is the first study evaluating outcomes with more than 14 years follow-up and the possible development of late complications after long periods of Ilizarov treatment for tibial comminuted fractures with bone loss and tibial deformities in a single-center and single-surgeon series.

Recently, May et al. evaluated closed tibial shaft fractures treated with the Ilizarov method and reported 59% chance of complications, 5% malunion, 1.3% deep infection and 6.5% persisting pin-site infection post-frame removal at a medium to long follow-up of 10 years [19]. Pin-track infection is the most common complication in using Ilizarov methods, with a rate between 10 and 100% among the studies analysed in a recent systematic review [5]. Based on our 30-year experience [10, 11, 20], we believe that this complication is so common in Ilizarov bone transport that it could be considered an unavoidable part of the treatment. For this reason, we decided to not consider it among the sequelae recorded at long follow-up.

Messner et al. reported that Ilizarov is a safe, effective, and reliable method for the treatment of tibial fractures with good but short-term outcomes [21]. Other studies have compared the complication rate of external fixation devices and highlighted possible solutions. However, these papers focus only on short-term complications or in the achievement of correction of the deformity [8, 22, 23]. In our cohort, during a mean clinical follow-up of 21.6 years, 56 out of 72 patients reported complications, and in 18 cases, there was a need for further surgical treatment under general anaesthesia. Among patients who reported complications, 36.1% (26/72 Pts) of them showed complications immediately after surgery and 55.6% (40/72 Pts) during the first 3 years. During the following years, complications were reported by 22.2% (16/72 Pts), and the last complications were reported 27 years after surgery for a patient who required a total knee arthroplasty for a type IIIb complication according to the Dindo Classification.

As expected, the majority of these sequalae referred to *FG*. Our data showed a direct correlation between neurological deficit, one of the most feared and permanent complications, and open fractures (*R* = 0.561, *p* < 0.001); and an inverse correlation between neurological deficit and LEFS (*R* = − 0.559, *p* < 0.001). Specifically, a direct correlation was found in the *FG* between neurological deficit and open fractures (R = 0.464, *p* = 0.013) and between EFT and open fractures (R = 0.495, *p* = 0.007). These poor clinical outcomes of the *FG* compared with the *DG* should be attributed to the severity of injuries included, 75% comminuted open fractures, most of them type IIIC (52.4%). These two aspects, bone and soft tissue loss, often associated to infection, determine a clinical situation so complex that even an amputation can be indicated, configuring the bone transport as a limb salvage procedure [1, 24]. In the review by Yin [17], the rate of amputation was 4%, which is similar to 2.9% reported by Papakostidis [18], 5.2% reported by Iliopoulous [25] and to 5.5% of our cohort, which however, has the longest follow-up. Hence, we believe that the apparent unsatisfied clinical results of the *FG* can be deemed good when clinical problems so critical are addressed without alternative solutions that can offer better results.

In the literature, alternative strategies to the Ilizarov method have been proposed for skeletal reconstruction, such as cancellous Masquelet style grafting [26]; vascularized bone grafts [27] and direct non-vascularized cancellous bone grafts [28]. None suites all case scenarios, as each technique has its place, indications, pros and cons. The Masquelet method should be considered an effective alternative procedure for femoral bone transport, which is frequently painful and a cause of severe knee stiffness in our experience. In agreement with recent authors [29], we think the induced membrane technique is less indicated for distal tibial bone defects where the membranes may not form significantly because of poor soft tissue coverage and blood supply. For bridging oncological resections and substantial bone loss beyond 10 cm, where segmental bone transport would necessitate a prolonged EFT, another reconstructive approach may be more appropriate, like a vascularised fibular graft [30]. However, in cases of tibial resections for bone and soft tissue loss, osteomyelitis or infected pseudoarthrosis, the surrounding soft tissues are almost always contaminated even if the site has been surgically restored, risking reinfection and failure of the vascularised graft implant. Song et al. [31] compared the results of bone transport and vascularised bone grafts in tibial defects, and the results were better in the bone transport group. Finally, when cancellous bone grafting is used alone for defects of > 5 cm, like those in our series, bone resorption occurs with a high failure rate [32]. Cierny and Zorn [33], comparing the results of treating segmental tibial defects using Ilizarov bone transport and massive autologous bone grafts, reported data in favour of the Ilizarov method.

In a recent narrative review [5], Aktuglu et al. reported the mean EFT for 619 patients of 27 studies was 10.7 months, while it was 9.19 months in Yin et al.’s study, which only included tibias [17], similar to the period reported in ours, 8.25 months. Our results show that complications are not related to EFT but seem to be related to the severity of the injury. Exposed fractures are predisposed to lower LEFS with respect to the group where Ilizarov was applied for closed tibial fracture or deformity correction. Although 22.2% of patients developed late complications (reduced range of motion, hip and knee pain) in our series, only 4 need further surgery for late infections due to previously exposed fractures that caused neurovascular impairment. The complication rates due to prolonged EFT, such as knee stiffness, malunion, refracture, infection and amputation have been reported to be 7%, 4%, 6%, 13%, 4% and 13%, respectively [5]. In our study, the overall complication rate was 77.8%: knee stiffness was found in 24 patients (33%), infection and amputation occurred in 4 cases (5.6%), while malunion and refracture were not recorded. However, as 58.3% of these complications were type I, no further surgical treatment was required. The high rate of complications in our series could be due to the mean lengthening of regenerated bone achieved, which is longer than is usually performed [9]. For this reason, most of the complications occurred during the first 3 years even if some sequelae arose even almost 30 years later.

Paley and Maar [34] suggested that double-level bone transport can be used when the bone defect is > 10 cm. Rozbruch et al. [35] subsequently modified this recommendation to > 6.0 cm for double level transport. All bone defects of our patients were > 4 cm and averaged 7.05 cm. As with the authors above, most of our patients (90%) received a single transport because most of the tibial defects were less than 6.5 cm. In Aktuglu’s review, the average number of cases in each study was 24 (range 7–86), while in ours, it was 72 cases. The mean follow-up duration was 34.05 months (range 6–122), compared to 259 months (range 180–360 months) reported in ours. Further, the authors referred to by Aktuglu [5] reported a complication rate per patient of 1.22, while our series revealed a lower rate of 0.9.

Bone grafting at the docking site is frequently necessary after bone transport is complete, often being recommended by different authors to avoid further complications [10, 36]. For this reason, it was carried out as a routine practice in our series. The ratio of bone union for Yin’s study [17], which only included tibias, was 96%, while for our patients it was higher (100%). Probably, this excellent result is due to the routine bone graft at the docking site. Because most of the tibial fractures of our cohort were open at the distal third (16/42), we believe that technically, the critical aspect of tibial bone transport occurs at this level, where it is possible to review the docking site and apply bone grafting to promote consolidation once the procedure is complete.

Several potential limitations and some biases may have influenced this case series study, mainly linked to its retrospective and single-center design. The primary limitation is the relatively low number of patients reached for the follow-up (less than 50%) compared to the overall number of patients treated. Nevertheless, our cohort is higher than those of other studies [17, 37]. Another limitation is the different bone conditions treated in the two groups analysed, however, with the same surgical technique used in both. When evaluating the Ilizarov bone transport method, most past studies have taken femur and tibia together into consideration, making it impossible to separate the clinical and functional data, often at short-medium periods. In contrast, our study focused only on tibial transport, both for fractures or deformities, showing outcomes and complications of a mono-center and a single-surgeon case series at long follow-up.

## Conclusions

Our results confirm that, even at a long follow-up period, Ilizarov bone transport is an effective technique for the definitive treatment of both of these challenging conditions: tibial comminuted fractures and tibial deformities. Functional outcomes, although significantly lower in patients with exposed fractures, are in line with those reported in the literature. However, they are not influenced by EFT when properly managed. Finally, orthopaedic surgeons should be aware that, although most complications occur during the first 3 years, they can also arise later, even up to nearly 30 years, as shown by our series.

## Data Availability

Any research materials of this study are available at our institution and can be accessed.
